# Association between DASH diet and asthma symptoms among a large sample of adolescents: a cross-sectional study

**DOI:** 10.1186/s40795-024-00884-4

**Published:** 2024-06-27

**Authors:** Vahid Arabi, Bahareh Sasanfar, Fatemeh Toorang, Zahra Nafei, Nasrin Behniafard, Amin Salehi-Abargouei

**Affiliations:** 1 Research Center for Food Hygiene and Safety, School of Public Health, Shahid Sadoughi University of Medical Sciences, Yazd, Iran; 2 Yazd Cardiovascular Research Center, Non-communicable Diseases Research Institute, Shahid Sadoughi University of Medical Sciences, Yazd, Iran; 3 Department of Nutrition, School of Public Health, Shahid Sadoughi University of Medical Sciences, Yazd, Iran; 4 Children Growth Disorder Research Center, Shahid Sadoughi University of Medical Sciences, Yazd, Iran; 5 Department of Allergy and Clinical Immunology, Shahid Sadoughi University of Medical Sciences, Yazd, Iran; 6 Cancer Research Center, Cancer Institute of Iran, Tehran University of Medical Sciences, Tehran, Iran; 7 Departments of Medical and Surgical Sciences, University of Bologna, Bologna, Italy; 8 Shahid Sadoughi Hospital, Ebne Sina Boulevard, Yazd, Iran; 9 Student Research Committee, Shahid Sadoughi University of Medical Sciences, Yazd, Iran

**Keywords:** DASH diet, Asthma, Asthma medication, Wheezing

## Abstract

**Background:**

The Dietary Approaches to Stop Hypertension (DASH) diet, which has a lot of emphasis on the consumption of fruits, vegetables, and whole grains, and on the other hand, the consumption of red meat and sodium is limited, due to its anti-inflammatory properties, which can be related to reducing the risk of asthma.

**Objectives:**

The aim of this study was to determine the relationship between the DASH diet and asthma symptoms among children and adolescents.

**Methods:**

This cross-sectional study was conducted among7667 children (3414 boys and 4253 girls) aged 6–7 and 13–14 years living in central Iran. Dietary food consumption was assessed using a multiple-choice questionnaire. Logistic regression was used to obtain odds ratios for the association between the DASH-like diet with current asthma and asthma symptoms.

**Results:**

Our findings revealed that higher adherence to a DASH-like diet resulted in lower odds of asthma confirmed by a doctor among the whole population (OR = 0.53; 95%CI: 0.36–0.76) and also in females (OR = 0.47; 95%CI: 0.29–0.78). Moreover, the higher adherence to the DASH-like diet was inversely associated with the chance of wheezing in the past 12 months in all subjects (OR = 0.67; 95%CI: 0.51–0.86) and in boys (OR = 0.57; 95%CI: 0.38–0.85).

**Conclusion:**

The findings of the present study showed that following the DASH diet can be associated with the improvement of asthma symptoms in children and adolescents. However, more research is needed to improve dietary recommendations for asthma prevention.

## Introduction

Asthma is a disease that causes many respiratory problems that affect modern societies and is known as inflammation or stenosis of the respiratory system [1]. It affects nearly 300 million people worldwide and 1,000 people per day pass away due to the illness. The prevalence of asthma has increased in the past 30 years, especially in industrialized countries where a large proportion of patients live [2]. According to statistics, one in 12 adults or 10 children worldwide suffer from asthma [3]. Asthma not only poses serious health risks such as increased mortality and disability, but also imposes significant financial and economic burdens on individuals and society [4]. According to statistics, the annual cost of asthma treatment in the U.S. is $81.9 billion, which means an average of $3,728 per person [5]. Based on the ISAAC questionnaire, more than 10% of Iranian children have asthma [6].

Common symptoms of asthma include wheezing, coughing, shortness of breath and fatigue [7]. Asthma is a multifactorial disease, and various factors such as genetics, drugs, environmental factors, and dietary intake play an essential role in its development or severity. Recognizing these factors can play a critical role in preventing, diagnosing, and treating this disease [8]. Diet is a key factor in influencing the occurrence or alleviation of asthma symptoms. Therefore, there has been a growing body of evidence on the link between dietary intake and asthma in recent years [9, 10]. For example, a healthy diet that emphasizes the consumption of fruits, vegetables, and antioxidants showed beneficial effects in reducing allergic rhinitis and asthma-like symptoms in children, while consuming foods such as margarine and processed meat worsened these symptoms [11, 12]. Epidemiological studies have reported a protective relationship between dietary antioxidants such as carotenoids, vitamin E, vitamin C, selenium and antioxidant-rich fruits with asthma [13]. Moreover, studies have indicated that eating fish at least once a week can help reduce the occurrence of asthma symptoms and lower the risk in children [14]. On the other hand, some interventional studies have shown disappointing results for the use of these antioxidants in asthma treatment [15–17].

The Dietary Approaches to Stop Hypertension (DASH) is an eating plan which emphasizes on receivinghighamountsoffruits, vegetables, low-fatdairyproducts, wholegrains, legumes, and nuts, and low amounts of consumption of red meat and processed meats, sodium consumption, and sweetened beverages [18]. Therefore, this dietary pattern is an antioxidant source diet (vitamins E, A, c and zinc), which might play a preventive role in asthma and respiratory problems by reducing the amount of malondialdehyde (MDA) and increasing glutathione (GSH) and generally reducing oxidative stress [19–21].

Few studies have assessed the relationship betweentheDash diet and asthma particularly in the Middle East.In such a way that, the results of these studies show the effectiveness of the Dash diet on asthma control [22]. Given the importance of asthma in adolescents and its impact on life, this study aimed to examine the association between the DASH diet and asthma symptoms among a large sample of Iranian adolescents.

## Subjects and methods

### Participants

This study was part of the Global Asthma Network (GAN) which was conducted in 2020 in one of the central cities of Iran. The GAN study is a multicenter cross-sectional study that suggests a minimum of 3,000 samples to accurately estimate the prevalence of asthma [23, 24].

Eighty-four schools from two educational districts of Yazd were randomly selected by using a cluster sampling design. The schools included both private and state elementary and high schools. We excluded non-Iranian people from the study. Due to the coronavirus pandemic and closures of schools, parents of 6–7 years old and subjects aged 13–14 answered an online questionnaire. Out of 7214 adolescents and 3026 children, 5141 and 2526 questionnaires were completed, respectively, and after reviewing the questionnaires, demographic data that were unacceptable were re-examined by telephone and necessary modifications were made if needed.

The Ethics Committee of Shahid Sadoughi University of Medical Sciences approved the study (IR.SSU.SPH.REC.1400.134). After that, the Yazd Education Department authorized the study in the relevant schools. A consent form was included at the beginning of the online questionnaires, and parents provided their consent for their children’s participation in the study.

### Asthma and its symptoms confirmation

The GAN questionnaire assesses the risk factors and symptoms of allergic diseases and this questionnaire has been extracted from ISACC questionnaire [25]. At first, the questionnaire was translated into Persian and then the reliability of the translated version was confirmed by a study conducted on 100 selected subjects by using Cronbach’s alpha. In this study, the alpha coefficient for asthma symptoms was estimated to be 0.862, indicating the reliability of this questionnaire. Finally, the questionnaire was translated into English and sent to GAN’s managers for approval.

In this study, participants were asked questions about asthma symptoms, asthma confirmed by a doctor, use of asthma medications, and frequent consumption of DASH diet ingredients over the past year. Based on the guidelines of this study, current asthma was defined as a history of confirmed asthma by a doctor and having had wheezing and/or use of asthma medication in the past 12 months.

### Assessment of dietary intakes

The frequency of dietary intake during the past year was evaluated by multiple choice questions in the GAN questionnaire [26]. Students were asked about the frequency of consumption of food groups such as fruits, vegetables, legumes, nuts, dairy, grains, meat, processed meats, sweets, and soft drinks, which are the main components of the DASH diet through food consumption frequency questionnaire.

### Assessment of the DASH-style diet score

The Dash diet constructed based on seven food components including high intake of fruits, vegetables, dairy, nuts and legumes, and grains and low intakes of Sweetened beverages, and red and processed meats [27]. In such a way that people who have the lowest consumption of components such as fruits, vegetables, dairy, nuts and legumes were placed in the first tertile and received a score of 1, and the people who had the highest consumption rate were placed in the third group and received a score of three. Those who were between the two groups in terms of consumption received a score of 2. We used an inverse method for grains, sweetened beverages, and red and processed meats; such that those in the third group of these food items were received a score of 1. In this study, because most of the grains consumed in Iran are refined grains [28], high consumption of this component we considered this group as a harmful dietary component.

### Assessment of other variables

In this study, the ethnicity of participants (Fars/Turk/Kurds/Arab/Baluch/Lur) height, weight and their use of computer and watching TV (2–4 h/5–8 h/9–14 h per day) were obtained using online self-reported questionnaire GAN. In addition, body mass index (BMI) was calculated using the following formula: weight (kg) divided by height squared (m^2^).

### Statistical methods

The Kolmogorov–Smirnov test was used to assess the normality. Individuals were categorized based on tertile of DASH scores. We used one-way ANOVA and chi-square tests to compare continuous and categorical variables, respectively, across tertile of DASH score. Multivariable logistic regression models were used to assess the association between adherence to the DASH-like diet and risk of asthma confirmed by a doctor, current asthma, usage of asthma medication, and wheezing in the last 12 months. The analyses were adjusted for age (continuous) and sex (girls, boys) first and additionally for watching TV & computer use (categorical) in model 2. We further controlled for BMI (continuous) in model 3. Pvalues < 0.05 were considered statistically significant. The analysiswas performed by STATA version 14 (State Corp., College Station, TX).

## Results

General characteristics of the subjects across tertiles of DASH-like diet intake are presented in Table 1. The gender was significantly different between the tertiles (P_value_=0.02). Higher DASH-like diet scores were associated with older age (P_value_<0.01). The frequency of ethnicity, physical activity, ever had wheezing and wheezing in the past 12 months was different among tertiles of DASH-like diet intake (P_value_<0.01).

**Table 1:** General characteristics of the subjects according to tertile of DASH-like diet intake

Variables	Tertile of DASH score			*P*-value
	T1	T2	T3	
Sex				
Male	1452 (46.1)	935 (44.5)	1027 (424)	**0.02**
Female	1696 (53.8)	1166 (55.5)	1391 (57.5)	
Age (years)	10.7 ± 3.4	11.0 ± 3.3	11.1 ± 3.2	**0.0001**
BMI (kg/m^2)^	18.7 ± 4.5	19.3 ± 18.1	18.9 ± 4.5	0.1
Ethnicity				**0.002**
Kord	14 (0.44)	5 (0.24)	24 (0.99)	
Turk	38 (1.21)	17 (0.81)	20 (0.83)	
Persian	3020 (95.9)	2034 (96.8)	2321 (95.9)	
Lor	23 (0.73)	19 (0.9)	24 (0.99)	
Arab	20 (0.64)	17 (0.81)	19 (0.79)	
Balooch	33 (1.05)	9 (0.43)	10 (0.41)	
Physical activity (watching TV and computer use)				**< 0.001**
2–4 h	1494 (47.4)	1167 (55.5)	1447 (59.8)	
5–8 h	1145 (36.3)	681 (32.4)	750 (31.0)	
9–14 h	509 (16.1)	253 (12.0)	221 (9.1)	
Asthma confirmed by a doctor				
Yes	138 (4.3)	96 (4.5)	90 (3.7)	0.31
No	3010 (95.6)	2005 (95.4)	2328 (96.2)	
Current asthma				
Yes	28 (1.01)	12 (0.64)	16 (0.72)	0.33
No	2744 (98.9)	1850 (99.3)	2196 (99.2)	
Use of asthma medication				
Yes	86 (2.7)	51 (2.4)	54 (2.2)	0.48
No	3062 (97.2)	2050 (97.5)	2364 (97.7)	
Ever had Wheezing				
Yes	576 (18.3)	352 (16.7)	328 (13.5)	**< 0.001**
No	2572 (81.7)	1749 (83.2)	2090 (86.4)	
Wheezing (in the past 12 months)				
Yes	294 (9.3)	167 (7.9)	148 (6.1)	**< 0.001**
No	2854 (90.6)	1934 (92.0)	2270 (93.8)	

Values are mean ± SD or number (percentages)

^a^χ^2^ Test for ordinal qualitative variables and T-test for continuous variables

Table 2 shows the frequency of food consumption of participants based on the tertile of DASH-Like diet score. The frequency consumption of fruits, vegetables, legumes, nuts, dairy, grains, processed meats, sweets, and beverages, and meat was significantly different between tertiles of DASH-Like diet score (P_value_< 0.01).

**Table 2:** Daily food of subjects according to tertile of DASH-like diet intake

Variables	Tertile of DASH score			*P*-value
	T1 (*n* = 3148)	T2 (*n* = 2101)	T3 (*n* = 2418)	
**Fruits**				
Never	248 (7.8)	16 (0.76)	4 (0.17)	**< 0.001**
Weekly	963 (30.5)	243 (11.5)	104 (4.3)	
Every day	1937 (61.5)	1842 (87.6)	2310 (95.5)	
**Vegetables**				
Never	1552 (49.3)	307 (14.6)	87 (3.6)	**< 0.001**
Weekly	1511 (48.0)	1494 (71.1)	1215 (50.2)	
Every day	85 (2.7)	300 (14.2)	1116 (46.1)	
**Legumes**				
Never	227 (7.2)	37 (1.7)	22 (0.91)	**< 0.001**
Weekly	2347 (74.5)	1288 (61.3)	831 (34.3)	
Every day	574 (18.2)	776 (36.9)	1565 (64.7)	
**Nuts**				
Never	1669 (53.0)	909 (43.2)	913 (37.7)	**< 0.001**
Weekly	1338 (42.5)	1006 (47.8)	1177 (48.6)	
Every day	141 (4.48)	186 (8.8)	328 (13.5)	
**Dairy**				
Never	164 (5.2)	13 (0.62)	8 (0.33)	**< 0.001**
Weekly	1454 (46.1)	452 (21.5)	232 (9.5)	
Every day	1530 (48.6)	1636 (77.8)	2178 (90.0)	
**Grains**				
Never	30 (0.95)	4 (0.19)	8 (0.33)	**< 0.001**
Weekly	395 (12.5)	279 (13.2)	438 (18.1)	
Every day	2723 (86.5)	1818 (86.5)	1972 (81.5)	
**Meat**				
Never	248 (7.8)	121 (5.7)	171 (7.0)	**< 0.01**
Weekly	1577 (50.1)	993 (47.2)	1156 (47.8)	
Every day	1323 (42.0)	987 (46.9)	1091 (45.1)	
**Process meats**				
Never	1506 (47.8)	1170 (55.6)	1632 (67.4)	**< 0.001**
Weekly	1454 (46.1)	860 (40.9)	769 (31.8)	
Every day	188 (5.9)	71 (3.3)	17 (0.7)	
**Sweets**				
Never	467 (14.8)	404 (19.2)	892 (36.8)	**< 0.001**
Weekly	1623 (51.5)	1253 (59.6)	1366 (56.4)	
Every day	1058 (33.6)	444 (21.1)	160 (6.6)	
**Beverages**				
Never	1423 (45.2)	1135 (54.0)	1698 (70.2)	**< 0.001**
Weekly	1253 (39.8)	821 (39.0)	678 (28.0)	
Every day	472 (14.9)	145 (6.9)	42 (1.7)	

Values are percentages

The association between tertiles of DASH-like diet score and asthma confirmed by a doctor for the total population, and subgroup analyses by age and sex, is provided in Table 3. A significant negative relationship was observed between risk of asthma and DASH-like diet score in the crude model, among the whole population [Odds ratio (OR):0.56, 95% confidence interval (CI): 0.39 to 0.80, P_trend_<0.01]. This relationship remained significant after adjustment for further confounders (OR:0.53, 95%CI: 0.36 to 0.76, P_trend_<0.001). Girls with highest adherence to DASH-like diet had a lowest odds for asthma confirmed by a doctor compared to those with lowest DASH-like diet score, in the crude model (OR: 0.52, 95%CI: 0.32 to 0.86, P_trend_= 0.01). This association was strengthened after controlling for further confounders (P_trend_< 0.01). There was no association between DASH-like diet score and asthma confirmed by a doctor among boys, in crude model, but after adjustment for further confounder, boys in higher tertile of DASH-like diet score had 41% decrease of asthma confirmed by a doctor (P_trend_=0.05). An inverse significant trend was found between DASH-like diet score and asthma confirmed by a doctor among 6–7 years old. In addition, children with highest adherence to DASH-like diet had a lowest odds of having asthma confirmed by a doctor compared to those with lowest DASH-like diet score among 13–14 years old in crude and full adjusted model (OR: 0.56, 95%CI: 0.37 to 0.85, P_trend_< 0.01).

**Table 3:** The association between DASH-like diet intake and Asthma confirmed by a doctor

DASH score				
	T1	T2	T3	*P*_trend_
	0R (95% CI)	0R (95% CI)	0R (95% CI)	
Girls				
Crude	1.00	0.87 (0.63–1.21)	0.52 (0.32–0.86)	**0.01**
Model 1	1.00	0.84 (0.60–1.17)	0.48 (0.29–0.78)	**0.005**
Model 2	1.00	0.84 (0.60–1.16)	0.47 (0.28–0.78)	**0.004**
Model 3	1.00	0.84 (0.60–1.17)	0.47 (0.29–0.78)	**0.004**
Boys				
Crude	1.00	0.84 (0.59–1.20)	0.60 (0.35–1.03)	0.06
Model 1	1.00	0.83 (0.58–1.18)	058 (0.34-1.00)	**0.04**
Model 2	1.00	0.83 (0.58–1.19)	0.59 (0.34–1.02)	**0.05**
Model 3	1.00	0.84 (0.58–1.19)	0.59 (035-1.02)	**0.05**
6–7 years old				
Crude	1.00	0.71 (0.42–1.19)	0.40 (0.15–1.02)	**0.03**
Model 1	1.00	0.72 (0.43–1.20)	0.40 (0.16–1.03)	**0.03**
Model 2	1.00	0.72 (0.43–1.20)	0.41 (0.16–1.06)	**0.03**
Model 3	1.00	0.72 (0.43–1.21)	0.41 (0.16–1.06)	**0.03**
13–14 years old				
Crude	1.00	0.87 (0.66–1.15)	0.56 (0.37–0.83)	**0.006**
Model 1	1.00	0.87 (0.66–1.15)	0.55 (0.37–0.83)	**0.006**
Model 2	1.00	0.87 (0.66–1.15)	0.55 (0.37–0.83)	**0.006**
Model 3	1.00	0.87 (0.66–1.15)	0.56 (0.37–0.85)	**0.006**
Total population				
Crude	1.00	0.86 (0.67–1.09)	0.56 (0.39–0.80)	**0.002**
Model 1	1.00	0.83 (0.65–1.06)	0.52 (0.36–0.75)	**0.001**
Model 2	1.00	0.84 (0.66–1.06)	0.52 (0.36–0.76)	**0.001**
Model 3	1.00	0.84 (0.66–1.06)	0.53 (0.36–0.76)	**0.001**

Model 1: adjusted for age and sex (for total participants)

Model 2: further adjusted for watching TV & computer use

Model 3: additionally, adjustment for BMI

There were no significant association between DASH-like diet intake and the likelihood of current asthma and asthma medication among whole population, girls, and boys (P_trend_> 0.05) (Tables 4 and 5**).** Table 6 provides information about the relationship between the score of the DASH-like diet and wheezing in the past 12 months. For the total population, in the crude model, individuals with the highest tertile of DASH-like diet score had a 33% lower wheezing chance compared to the lowest tertile (OR: 0.67, 95%CI: 0.52 to 0.86, P_trend_<0.01). Also, after adjustment for more confounders this relation remained significant (OR: 0.67, 95%CI: 0.51 to 0.86, P_trend_<0.01). In addition, we found that boys in the top tertile of DASH-like diet had lower odds of wheezing in the past 12 months, compared with those in the bottom tertile (OR: 0.57, 95%CI: 0.38 to 0.85, P_trend_<0.01). For girls, we did not find significant association in crude and full models. However, girls in top tertile of DASH-like diet had 28% lower risk of wheezing in the past 12 months, compared with those in the bottom tertile, when adjusted for age and energy.

**Table 4:** The association between DASH-like diet intake and likelihood of current asthma

DASH score	T1	T2	T3	*P* _trend_
	0R (95% CI)	0R (95% CI)	0R (95% CI)	
Girls				
Crude	1.00	0.77 (0.31–1.89)	0.19 (0.02–1.46)	0.09
Model 1	1.00	0.8 (0.32–1.96)	0.21 (0.02–1.64)	0.13
Model 2	1.00	0.79 (0.32–1.95)	0.2 (0.02–1.57)	0.11
Model 3	1.00	0.79 (0.32–1.96)	0.2 (0.02–1.56)	0.11
Boys				
Crude	1.00	1.00 (0.49–2.04)	0.63 (0.21–1.90)	0.5
Model 1	1.00	1.05 (0.51–2.15)	0.70 (0.23–2.10)	0.64
Model 2	1.00	1.06 (0.52–2.17)	0.71 (0.23–2.15)	0.67
Model 3	1.00	1.06 (0.52–2.17)	0.71 (0.23–2.15)	0.68
Total population				
Crude	1.00	0.89 (0.51–1.57)	0.42 (0.16–1.09)	0.1
Model 1	1.00	0.94 (0.54–1.65)	0.48 (0.18–1.25)	0.19
Model 2	1.00	0.94 (0.54–1.65)	0.48 (0.18–1.25)	0.19
Model 3	1.00	0.94 (0.54–1.65)	0.48 (0.18–1.25)	0.19

Model 1: adjusted for age and sex (for total participants)

Model 2: further adjusted for watching TV & computer use

Model 3: additionally, adjustment for BMI

**Table 5:** The association between a DASH-like diet intake and Use of asthma medication

DASH score				
	T1	T2	T3	*P*_trend_
	0R (95% CI)	0R (95% CI)	0R (95% CI)	
Girls				
Crude	1.00	0.79 (0.46–1.35)	1.12 (0.61–2.04)	0.92
Model 1	1.00	0.78 (0.46–1.34)	1.10 (0.60–2.01)	0.96
Model 2	1.00	0.78 (0.46–1.34)	1.11 (0.60–2.03)	0.94
Model 3	1.00	0.78 (0.46–1.34)	1.10 (0.60–2.02)	0.95
Boys				
Crude	1.00	0.94 (0.63–1.40)	0.78 (0.44–1.37)	0.42
Model 1	1.00	0.92 (0.61–1.37)	0.75 (0.42–1.32)	0.33
Model 2	1.00	0.93 (0.62–1.38)	0.76 (0.43–1.34)	0.37
Model 3	1.00	0.93 (0.62–1.38)	0.76 (0.43–1.34)	0.37
Total population				
Crude	1.00	0.87 (0.63–1.20)	0.89 (0.59–1.34)	0.48
Model 1	1.00	0.87 (0.63–1.19)	0.89 (0.59–1.34)	0.46
Model 2	1.00	0.87 (0.63–1.20)	0.90 (0.60–1.36)	0.5
Model 3	1.00	0.87 (0.63–1.20)	0.9 (0.59–1.36)	0.5

Model 1: adjusted for age and sex (for total participants)

Model 2: further adjusted for watching TV & computer use

Model 3: additionally, adjustment for BMI

**Table 6:** The association between a DASH-like diet intake and wheezing in the past 12 months

DASH score				
	T1	T2	T3	*P*_trend_
	0R (95% CI)	0R (95% CI)	0R (95% CI)	
Girls				
Crude	1.00	0.89 (0.69–1.15)	0.75 (0.53–1.04)	0.09
Model 1	1.00	0.88 (0.68–1.13)	0.72 (0.51-1.00)	**0.05**
Model 2	1.00	0.89 (0.69–1.15)	0.75 (0.54–1.06)	0.1
Model 3	1.00	0.89 (0.69–1.15)	0.75 (0.54–1.06)	0.1
Boys				
Crude	1.00	0.84 (0.65–1.09)	0.59 (0.40–0.87)	**0.008**
Model 1	1.00	0.81 (0.62–1.05)	0.55 (0.37–0.81)	**0.002**
Model 2	1.00	0.82 (0.63–1.07)	0.57 (0.39–0.85)	**0.005**
Model 3	1.00	0.82 (0.63–1.07)	0.57 (0.38–0.85)	**0.005**
Total population				
Crude	1.00	0.86 (0.72–1.04)	0.67 (0.52–0.86)	**0.002**
Model 1	1.00	0.84 (0.70–1.01)	0.64 (0.49–0.82)	**0.000**
Model 2	1.00	0.86 (0.71–1.03)	0.67 (0.52–0.86)	**0.002**
Model 3	1.00	0.86 (0.71–1.03)	0.67 (0.51–0.86)	**0.002**

Model 1: adjusted for age and sex (for total participants)

Model 2: further adjusted for watching TV & computer use

Model 3: additionally, adjustment for BMI

## Discussion

As far as we know, no previous study has examined the link between DASH diet and asthma symptoms among adolescents. Due to the lack of data on sodium intake, we were unable to incorporate it into the score calculation. Therefore, we have provided a DASH-like diet score without considering sodium intake. Our findings revealed that higher adherence of DASH-like diet score resulted in lower odds of asthma confirmed by a doctor among whole population and subgroup analysis by sex. Moreover, the higher adherence to the DASH-like diet was inversely associated with the chance of wheezing in all adolescents, girls and boys.

The DASH diet is very similar to the Mediterranean diet in terms of its components. Luis Garcia-Marcos et al., in their cross-sectional study on schoolchildren, showed the protective effects of each Mediterranean score unit with current severe asthma in girls (adjusted OR 0.90, 95% CI 0.82 to 0.98) [29]. They also showed the protective effects of seafood and cereals for severe asthma, while fast food was a risk factor [29]. Another cross-sectional study on children revealed that greater adherence to the Mediterranean diet was negatively associated with ever diagnosed asthma [30]. A similar relationship was found in another cross-sectional study, as well [31]. Contrary to our results, in a population-based case-control study conducted by Bakolis et al. in 2010, no relationship was observed between a prudent diet (whole meal bread, fish, and vegetables) and asthma [32]. In addition, a study on a large population of adult French women (Varraso et al., 2009) did not observe any relationships between a prudent pattern, a Western pattern, and nuts and wine pattern with the incidence of asthma, ever asthma, or current asthma. They just found a lower frequency of asthma with nuts and wine consumption in the highest tertile (OR: 0.65; 95% CI: 0.31 to 0.96), and a higher frequency of asthma with the Western dietary pattern (OR: 1.79; 95% CI: 1.11 to 3.73) [33]. The present study differs from previous studies in several ways, such as the difference in sample size and a more comprehensive examination of different genders and a wider age range. Unlike in Iran, where wine consumption is prohibited due to religious reasons, and the diet is mostly composed of carbohydrate-rich, economical foods, the Western diet is more prevalent in the societies studied in previous research.

The current study showed that a DASH-like diet is inversely associated with asthma and wheezing. Consistent with our findings, previous studies have shown that a DASH diet has beneficial effects on asthma and asthma symptoms [34, 35]. Some studies have evaluated the effects of various food groups in the DASH diet on the risk of asthma and its symptoms. According to a meta-analysis by Rezazadeh et al., there is an inverse relationship between the intake of fruits and vegetables and asthma symptoms [36]. Moreover, A prospective cohort study by Papadopoulou et al. revealed an association between fruit and vegetable consumption and lower risk of asthma symptoms [37]. Other studies confirmed the results of this study; In a cross-sectional study, an inverse relationship was observed between fruit consumption ≥ 3 times/week and asthma wheeze and severe asthma symptoms among children aged 6–7 years and adolescents [38].

Previous studies have shown that the DASH diet, which consists of low-fat dairy products, legumes, vegetables, and B-carotene, can lower the risk of asthma by reducing inflammation and pro-inflammatory markers such as interleukin-6 (IL-6) and tumor necrosis factor alpha (TNF-α) levels [39–41]. As TNF is a biomarker for severe asthma, which leads to the remodeling of smooth muscle, engagement of immune cells, and induction of chronic inflammation in the airways, anti-TNF agents are considered therapeutics for patients with severe asthma [42]. Moreover, IL-6 is involved in airway remodeling during asthma and maintains chronic inflammation in the respiratory tract according to research on human bronchial tissue samples. IL-6 increases Th2-associated cytokine production, initiates Th17-cell differentiation, inhibits Th1-cell expansion, and suppresses Treg cells. An increase in T-helper cells (Th2 or Th1/Th17) expands the infiltration of granulocytes into the airways, which leads to the release of pro-inflammatory cytokines and subsequent inflammation [42]. Therefore, dietary patterns with anti-inflammatory characteristics, such as the DASH diet, may play a role in preventing asthma.

The high content of antioxidants, including vitamin C, vitamin E, and β-carotene, in the DASH diet might also play an important role in reducing asthma risk [43–45]. The lack of balance between reactive oxygen species (ROS) and antioxidants leads to oxidative stress, which can exacerbate asthma by increasing inflammation [46]. Vitamin C supports the hydration of airway surfaces and decreases free radical levels [47]. Evidence has demonstrated antioxidant and anti-inflammatory effects of vitamin E on airway inflammation or injury [48, 49]. In addition, vitamin E interrupts lipid peroxidation and prevents oxidant-induced membrane damage [50]. β-Carotene can reduce the highly reactive free radical superoxide anion and reacts with peroxyl free radicals [51].

The DASH diet has a high fiber content, which can play a role in reducing the risk of asthma and wheezing. A cohort study by Andrianasolo et al. on French adults showed a protective effect of fiber on asthma [52]. In a study by Saeed et al., a high-fiber diet was associated with a lower prevalence of current asthma in adults [53]. A potential mechanism for explaining the anti-inflammatory effect of fiber is the increased production of circulating short-chain fatty acids (SCFAs) formed after fiber fermentation by the gut microbiota [54, 55]. SCFAs can reduce the pulmonary response to inflammatory stimuli through activation of free fatty acid receptors [56, 57]. The results of the present study also confirm this issue, in that greater adherence to the DASH diet, which is a rich source of anti-inflammatory compounds, antioxidants, and high fiber content, was associated with a reduction in the risk of asthma confirmed by a doctor and the presence of wheezing.

This study had several strengths and limitations. To the best of our knowledge, this is the first study to evaluate the association between a DASH diet and asthma among adolescents. In addition, a large sample size, adjustment for multiple potential confounders, and conducting stratified analyses are the strengths of this study. Our study has several potential limitations that should be considered before interpreting its results. Firstly, the cross-sectional nature of this study does not imply a cause-and-effect association. Secondly, the data of our study were collected from self-reported questionnaires, which are prone to biases. Thirdly, estimation of dietary intake using the FFQ can lead to misclassification and misreporting.

## Conclusion

In conclusion, the findings of the current study showed that following the DASH diet can be associated with the improvement of asthma symptoms in children and adolescents. However, more research is needed to improve dietary recommendations for asthma prevention.

## Data Availability

The datasets generated and/or analysed during the current study are available from the corresponding author on reasonable request.
